# Hydrogen Uptake and Embrittlement of Carbon Steels in Various Environments

**DOI:** 10.3390/ma13163604

**Published:** 2020-08-14

**Authors:** Anton Trautmann, Gregor Mori, Markus Oberndorfer, Stephan Bauer, Christoph Holzer, Christoph Dittmann

**Affiliations:** 1 Department General, Analytical and Physical Chemistry, Montanuniversitaet Leoben, Franz-Josef-Strasse 18, 8700 Leoben, Austria; anton.trautmann@unileoben.ac.at (A.T.); mori@unileoben.ac.at (C.H.); 2 Green Gas Technology, RAG Austria AG, Schwarzenbergplatz 16, 1015 Vienna, Austria; markus.oberndorfer@rag-austria.at (M.O.); stephan.bauer@rag-austria.at (S.B.); 3 Research & Development, voestalpine Tubulars GmbH & Co KG, Alpinestrasse 17, 8652 Kindberg, Austria; christoph.dittmann@vatubulars.com

**Keywords:** hydrogen embrittlement, hydrogen absorption, hydrogen gas, sour gas, autoclave tests

## Abstract

To avoid failures due to hydrogen embrittlement, it is important to know the amount of hydrogen absorbed by certain steel grades under service conditions. When a critical hydrogen content is reached, the material properties begin to deteriorate. The hydrogen uptake and embrittlement of three different carbon steels (API 5CT L80 Type 1, P110 and 42CrMo4) was investigated in autoclave tests with hydrogen gas (H_2_) at elevated pressure and in ambient pressure tests with hydrogen sulfide (H_2_S). H_2_ gas with a pressure of up to 100 bar resulted in an overall low but still detectable hydrogen absorption, which did not cause any substantial hydrogen embrittlement in specimens under a constant load of 90% of the specified minimum yield strength (SMYS). The amount of hydrogen absorbed under conditions with H_2_S was approximately one order of magnitude larger than under conditions with H_2_ gas. The high hydrogen content led to failures of the 42CrMo4 and P110 specimens.

## 1. Introduction

When steels are exposed to hydrogen, their mechanical properties can deteriorate. This phenomenon is commonly referred to as hydrogen embrittlement and has been studied for 145 years [1]. Sir Harry Bhadeshia emphasized that one of the key elements in understanding and preventing hydrogen embrittlement is knowing the amount of hydrogen that a material absorbs under certain conditions [2]. In dry conditions, i.e., without electrochemical reactions such as corrosion, the only possible source for absorbable hydrogen atoms is the hydrogen gas (H_2_). The hydrogen molecule dissociates to two adsorbed hydrogen atoms (H_ad_) on the steel surface. H_ad_ can subsequently be absorbed by the material (H_ab_). The hydrogen solubility of metal (S) is depicted in Sieverts‘ law [3]:

(1) $$S=S_{0}\cdot \sqrt{p}\cdot e^{-\frac{\Delta H}{R\cdot T}}$$

where S_0_ is the solubility constant, p is the partial pressure of the H_2_ gas, ΔH the heat of solution, R the universal gas constant and T the absolute temperature. The equation expresses the pressure and temperature dependence of the hydrogen solubility as first described by Sieverts and Krumbhaar [4].

In conditions with electrolyte, corrosion of steel can be a second potential source for hydrogen. The electrons, that are released when the metal is dissolved, reduce the protons (H^+^) present in the electrolyte to hydrogen atoms. If some of the hydrogen atoms do not recombine to molecules, they can get adsorbed and further absorbed by the material. It is known in the oil and gas industry that hydrogen sulfide (H_2_S) can cause many problems, such as sulfide stress cracking (SSC). H_2_S hinders the recombination of hydrogen atoms and therefore promotes their absorption [5,6].

The crucial role of the microstructure on hydrogen solubility and thus the possible hydrogen uptake of a material was shown by Siegl et al. [7] in permeation experiments. The difference in the solubility of pure iron between the recrystallized condition and the severely plastically deformed material was a factor of approximately 4000. Hydrogen traps and consequently the local hydrogen concentration strongly influence diffusion [8,9].

To initiate a crack three things have to come together: a critical hydrogen concentration, a certain stress level and a susceptible material [10]. In addition to strength [11], microstructure strongly influences the susceptibility of a material to SSC and to hydrogen embrittlement [12]. Tempered martensite generally shows good resistance to SSC [13], but sensitivity increases with increasing strength [11,14]. The volume fraction of tempered martensite has to be as high as possible [15]. A minimum martensite fraction of 95% is standardized for high strength sour service grades [16]. Retained austenite significantly increases the hydrogen solubility and decreases ductility of a hydrogen charged martensitic steel [17]. Failure due to hydrogen embrittlement can occur particularly when strain-induced martensite forms in austenite grains [18]. The freshly formed martensite then has a high concentration of hydrogen, which considerably facilitates cracking [19].

Thresholds for hydrogen contents that cause hydrogen embrittlement of specific steel grades are rare in the literature. Values for the amount of hydrogen absorbed in H_2_ gas at medium to high pressures are even more difficult to find. Nevertheless, several values for various steel grades and hydrogen charging conditions could be obtained from literature and are summarized in this publication.

Asahi et al. [20] found a critical hydrogen content of around 2.5 wt. ppm and 0.4 wt. ppm for martensitic steels with an ultimate tensile strength (UTS) of 1062 MPa and 1212 MPa, respectively. Based on their results, they proposed equations to calculate the critical hydrogen concentration of any oil country tubular goods (OCTG) grade. For the martensitic steel grades L80 and P110, they calculated threshold hydrogen contents of 22.8 wt. ppm and 1.3 wt. ppm for embrittlement at room temperature. A yield strength (YS) of 765 MPa results in a value of 8.2 wt. ppm. They also proposed an increasing hydrogen embrittlement threshold with increasing temperature. Peral et al. [21] tempered samples of the martensitic steel 42CrMo4 at two different temperatures and charged them with 195 bar H_2_ gas at 450 °C for 21 h. The material tempered at a lower temperature had a YS of 1086 MPa, an UTS of 1200 MPa and absorbed 1.9 wt. ppm hydrogen. The specimens tempered at a higher temperature (YS = 622 MPa, UTS = 710 MPa) had a total hydrogen content of 1.2 wt. ppm after charging. Despite the high hydrogen concentration, the tensile properties of smooth specimens that were tested with slow strain rates remained practically unaffected. Tensile tests on notched specimens, however, showed a significant decrease in the UTS of the higher strength material. Hüter et al. [22] conducted slow strain rate tests on hydrogen charged, martensitic X20Cr13 with an UTS of 1046 MPa. At a hydrogen content of 5 wt. ppm they observed a brittle behaviour, while 1.5 wt. ppm (uncharged condition) resulted in a fracture surface with fully ductile features. Wang et al. [23] conducted constant load tests on notched AISI 4135 (34CrMo4) martensitic steel specimens that had previously been electrochemically charged. Tempering at two different temperatures had resulted in yield strengths of 1235 MPa and 1320 MPa and ultimate tensile strengths of 1320 MPa and 1450 MPa. The higher strength material had a critical hydrogen concentration of 0.06 wt. ppm, the lower strength 0.2 wt. ppm. The load was 90% of the UTS. They found that the critical hydrogen concentration not only depends on the strength level, but also on the stress concentration factor determined by the geometry of the notch. The same authors tested un-notched tensile specimens of the lower strength material and found no significant change in the UTS, while the fracture elongation was reduced at a hydrogen content of 1.2 wt. ppm [24].

Todoshchenko et al. [25] conducted slow strain rate tests on hydrogen charged ASTM A106 Grade B (UTS ≅ 420 MPa). A hydrogen content of 1.11 wt. ppm resulted in a slightly increased tensile strength and a significantly reduced fracture elongation of the ferritic-pearlitic material. Revie et al. [26] tested various ferritic-bainitic linepipe steels in H_2_S-saturated buffered solution and found threshold hydrogen concentrations of 0.2 to 1.8 wt. ppm for hydrogen-induced cracking. Unfortunately, they did not specify the strength of the steel grades examined. Kittel et al. [27] performed HIC tests on linepipe steels similar to API 5L X65. Based on the results for five materials (YS ≅ 530 MPa, UTS ≅ 632 MPa) they proposed a critical hydrogen concentration of 0.8 to 1 wt. ppm for conventional ferritic-pearlitic steels. Others [28] found a threshold of 0.26 wt. ppm for the ferritic-pearlitic linepipe steel API 5L X60 (YS = 414 MPa). They performed HIC tests with H_2_S and calculated the hydrogen content from permeation measurements. Kappes et al. [29] conducted permeation experiments on X65 in an H_2_S-saturated solution. A surface concentration of around 0.58 wt. ppm hydrogen significantly increased the fatigue crack growth rate of the steel (YS = 471 MPa). Hara et al. [30] proposed a critical hydrogen concentration of 0.3 to 2 wt. ppm for hydrogen induced cracking (HIC) initiation in X65 linepipe steel. They found differences in the behaviour of pearlitic and bainitic microstructures, the latter being much more resistant. Wang [31] found a critical hydrogen concentration of approximately 1 wt. ppm for the linepipe steel API X70. Above this limit, the fracture toughness of the material (YS = 583 MPa) consisting of ferrite and degenerated pearlite decreases in a linear relationship with increasing hydrogen content. In so called small punch tests hydrogen gas with a pressure of only 0.5 bar is sufficient to trigger brittle behaviour of the ferritic-bainitic X70 pipeline steel [32]. The severe stress concentration conditions prevailing in these tests make it easier to reach the local critical hydrogen concentration. 3.6 wt. ppm of hydrogen add an additional tensile stress to X80 linepipe steel under external tension and therefore facilitate plastic deformation [33]. Meng et al. [34] performed slow strain rate tests on X80 pipeline steel (YS = 524 MPa, UTS = 656 MPa) in H_2_/N_2_ mixtures at room temperature. While the strength and fracture elongation of smooth tensile specimens remained largely unaffected by the hydrogen gas up to a pressure of 60 bar, they observed a drop in the reduction of area between a hydrogen partial pressure of 12 and 24 bar. Six bar of H_2_ gas was sufficient to dramatically degrade the fatigue life. Even a hydrogen pressure of 300 bar did not influence the YS and the UTS of the ferritic-pearlitic X80 pipeline steel in slow strain rate tests, while a decrease of the fracture elongation was observed [35]. The embrittlement of the material (YS = 510 MPa) started at 1 bar, increased until a pressure of 50 bar and then stabilized [36]. Nagao et al. [37] charged the tempered martensitic steel API 5CT Q125 in 400 bar H_2_ gas at 200 °C. They measured a hydrogen content of 0.8 wt. ppm in the material (YS = 911 MPa, UTS = 1001 MPa).

The pressure vessel steel 2.25Cr-1Mo absorbs 3 wt. ppm hydrogen when charged in 120 bar H_2_ gas at 420 °C [38]. The threshold for hydrogen embrittlement of the material (YS = 475 MPa, UTS = 614 MPa) was found at 1.6 wt. ppm. Al-Rumaih and Gangloff [39] found a threshold of 1.4 wt. ppm for the bainitic 2.25Cr-1Mo (YS ≅ 530 MPa). No deterioration in the fracture resistance was observed below this value. Above the threshold it decreases dramatically [40]. A vanadium modified Cr-Mo steel of similar strength resists hydrogen embrittlement up to a hydrogen concentration of approximately 9 wt. ppm [41]. Álvarez et al. [42] charged the bainitic-martensitic 2.25Cr1Mo0.3V (YS = 590 MPa, UTS = 700 MPa) in 195 bar H_2_ gas at 450 °C and measured a hydrogen content of 4.2 wt. ppm. The fracture surface of the single-edge notched tensile specimen was dominated by ductile features. Venezuela et al. [43] charged the 3.5NiCrMoV with 2, 20, 80 and 200 bar of H_2_ gas, which resulted in a hydrogen content of 0.57, 0.64, 0.87 and 0.89 wt. ppm. The charging took 24 h, the first two hours at 200 °C and then cooling to room temperature in the turned off furnace. The quenched-and-tempered, martensitic steel had a YS of 661 MPa and an UTS of 782 MPa. Even electrochemically generated hydrogen concentrations of up to 1.93 wt. ppm, which correspond to hydrogen fugacities of over a thousand bar, did not affect the ductility of this steel when tested in linearly increasing stress tests, although brittle areas were observed on the fracture surface [43,44]. With cathodic charging, AISI 4340 (36CrNiMo4) has a saturation value of approximately 5 to 8 wt. ppm [45,46]. In 1.1 bar H_2_ gas at room temperature Bandyopadhyay et al. [47] calculated a hydrogen content of 0.01 wt. ppm for the martensitic steel (YS = 1138 MPa). Das et al. [48] simulated hydrogen charged notched AISI 4340 bars (YS = 1270 MPa) in four-point bending tests and found a threshold hydrogen concentration for the brittle to ductile transition of 0.02 wt. ppm. Garrison and Moody [49] reported decreasing threshold stress intensity factors with increasing hydrogen pressure and increasing YS of AISI 4340.

Based on the research of Scharf et al. [50] a hydrogen content of approximately 1.2 wt. ppm is sufficient to cause embrittlement of a ferritic-martensitic high-strength dual-phase steel that is loaded at 80% of its YS. A hydrogen content of 1.5 wt. ppm causes a significant deterioration of the fracture elongation of a quenching & partitioning treated high strength steel (YS = 746 MPa, UTS = 1013 MPa) [51]. Lovicu et al. [19] investigated two martensitic high-strength steels (YS = 1205 MPa, UTS = 1305 MPa and YS = 1410 MPa, UTS = 1520 MPa). They reported a critical hydrogen concentration of about 4 wt. ppm for the former and about 1 wt. ppm for the latter. Kim et al. [52] found a clear dependence of the susceptibility to hydrogen embrittlement of high-strength steels on the microstructure. For a fully pearlitic steel with an UTS of slightly over 1600 MPa they found a critical hydrogen content of around 0.42 wt. ppm. The same steel with a tempered martensitic microstructure and the same strength showed hydrogen embrittlement from a hydrogen concentration of 0.07 to 0.20 wt. ppm. Thomas et al. [53] reported a significant and continuous decrease in threshold stress intensity of an ultrahigh-strength martensitic steel (YS = 1765 MPa, UTS = 1985 MPa) between 0.3 wt. ppm (uncharged condition) and 10 wt. ppm. When loaded with its YS of more than 2000 MPa, the bainitic high-strength steel UNS G10700 (Ck67) shows hydrogen embrittlement from a hydrogen content of 1.5 wt. ppm. For the molybdenum-modified version with same strength and microstructure, 0.7 wt. ppm hydrogen are sufficient to cause cracking [54].

The ferritic-austenitic duplex stainless steel 2205 absorbs 0.83 wt. ppm hydrogen when charged in 100 bar H_2_ gas at 80 °C. The addition of an aqueous electrolyte results in a significantly higher uptake. Nevertheless, with a constant load of 90% of the specified minimum yield strength (SMYS), the hydrogen content is not sufficient to cause embrittlement [55]. Takai et al. [56] charged the austenitic stainless steel SUS 316L in hydrogen gas with various pressures at 90 °C. 100 bar resulted in a hydrogen content of 9 wt. ppm, 200 bar in 20 wt. ppm and 450 bar in 39 wt. ppm. The amount of hydrogen absorbed by austenitic steels is not only significantly higher than the amount absorbed by carbon steels, but also the amount of hydrogen required to initiate embrittlement is one or two orders of magnitude greater than that of steels with bcc lattice [57].

The aim of the presented work is to generate data on the hydrogen uptake of three carbon steels under seven different conditions at two temperatures and to check whether hydrogen embrittlement occurs.

## 2. Materials and Methods

Carbon steels that are commonly used in the oil and gas industry were investigated: L80 Type 1, P110 (both according to API 5CT [16]) and 42CrMo4 (UNS G41400). Samples were taken from commercially available casing tube sections. The chemical composition of the materials investigated is given in Table 1.

The mechanical properties of the tested materials are given in Table 2. The values were obtained in tensile tests performed in air on small, non-standard tensile specimens with an initial gauge length of 25 mm and a circular cross section with a diameter of 3 mm. The strain rate was 6.7 × 10^−5^ s^−1^.

**Figure 1:**
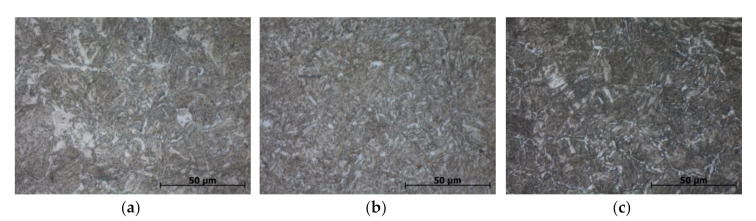
Microstructure of the investigated materials: (**a**) L80; (**b**) 42CrMo4; (**c**) P110.

The investigated materials can be assigned to three different strength levels, with L80 having the lowest yield strength, P110 having the highest and 42CrMo4 lying in the middle. The ultimate tensile strength is the same for 42CrMo4 and P110, while the L80 is lower. Figure 1 shows the microstructure of the examined steel grades.

All three materials have a microstructure of tempered martensite. Former austenite grains have a size of 20 to 40 µm, which is fine. The microstructure of 42CrMo4 is somewhat finer than that of the other two materials. The volume fraction of retained austenite was determined by means of X-ray diffraction with Mo Kα radiation in a ‘Bruker D8 DISCOVER’ in accordance with ASTM standard E975-13 [58]. The samples were wet ground with silicon carbide paper (grit 120, 320 and 500) and a 150 µm thick layer was electrochemically removed prior to analysis. The amount of retained austenite in all three materials was below the detection limit of 1% specified in the standard. Two types of specimens were tested: Immersion specimens were cuboids with a square base (edge length of 6 mm) and a height of 30 mm. The small tensile specimens for the constant load tests (CLTs) were the same as for the tensile tests in air mentioned earlier. The load of 90% of the specified minimum yield strength (SMYS) was applied to the respective CLT specimen with a spring made of a cobalt-base alloy and ceramic nuts, the latter ensuring electronic decoupling of the specimen from the more noble spring. All specimens were tested in the as-machined condition. Prior to testing, the specimens were cleaned in an ultrasonic bath with acetone.

**Figure 2:**
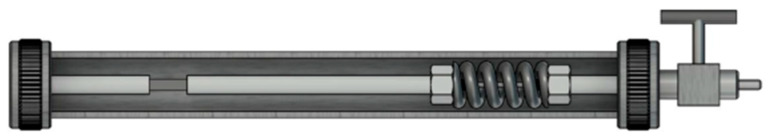
Experimental setup of the autoclave tests (schematic).

Two different types of tests were conducted: autoclave tests at elevated pressure and tests at ambient pressure. For the former, the installation of specimens within the autoclave is shown schematically in Figure 2.

**Figure 3:**
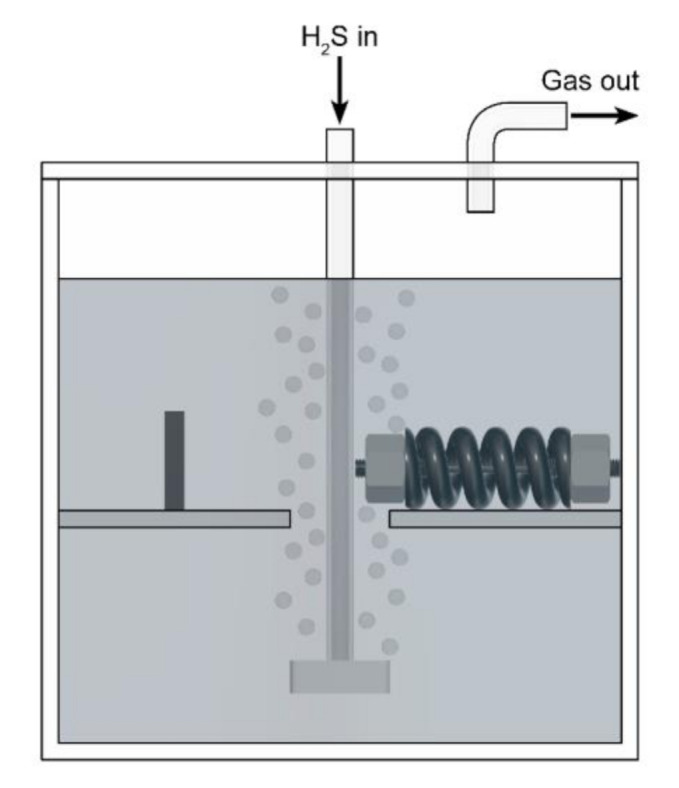
Experimental setup of the tests at ambient pressure (schematic).

The immersion specimen (left) and the CLT specimen (right) were held in place by polytetrafluoroethylene (PTFE) parts. Tests were conducted under anaerobic conditions with H_2_ gas at partial pressures of 20 and 100 bar. In addition, the influence of 5 bar CO_2_ gas was investigated. The autoclave tests were divided into tests with and without electrolyte, which are referred to as wet and dry conditions. The electrolyte (brine) was an aqueous NaCl solution with a chloride concentration of 15,000 ppm. The tests were carried out at 25 °C as well as 80 °C and lasted 30 days. The autoclaves were rotated with a speed of 1 RPM. Consequently, the specimens were periodically wetted with the brine, if any. The test procedure is described in detail elsewhere [59,60]. The experimental setup of the tests at ambient pressure is shown schematically in Figure 3. The geometry and handling of the specimens was the same as for the autoclave tests. The medium for the tests at ambient pressure was the ‘Solution A’ described in the NACE standard TM0177 [61]. This is an acidified H_2_S-saturated aqueous brine solution with 5.0 wt.% NaCl and 0.5 wt.% CH_3_COOH. The tests were conducted at room temperature and lasted 14 days.

After the autoclave tests, the immersion specimens were removed from the vessels and immediately cooled in liquid nitrogen. Specimens immersed at ambient pressure were removed after different times of charging in H_2_S and instantly cooled in liquid nitrogen too. The cooled specimens were ground with silicon carbide paper (grit 120) to remove corrosion products. The sides of the specimens were ground individually and after grinding each side of the specimens they were again cooled in liquid nitrogen. The specimens were never outside the liquid nitrogen for more than 15 s at a time. Before the hydrogen analysis was done, each specimen was rinsed with acetone, dried with a cold air fan, and weighed. The time between the removal from the liquid nitrogen and the start of the analysis in the furnace was less than 60 s. The hydrogen content was measured in an ELTRA H-500 (ELTRA GmbH, Haan, Germany) analyser after carrier gas hot extraction at 950 °C. The extracted hydrogen results in an increase of thermal conductivity of the carrier gas (pure nitrogen), expressed as a voltage peak as a function of time. The integral of the peak is a measure of the hydrogen extracted from the specimen. Calibration was done twice with calibration gas and gold-coated calibration standards. At the end of the autoclave tests and periodically during the H_2_S tests, the constant load specimens were checked for possible fractures.

## 3. Results

Each material was tested under seven different conditions. Selected conditions were tested up to three times. Since a maximum deviation of ± 15% was found, all error bars show this value.

### 3.1. Hydrogen Analyses

**Figure 4:**
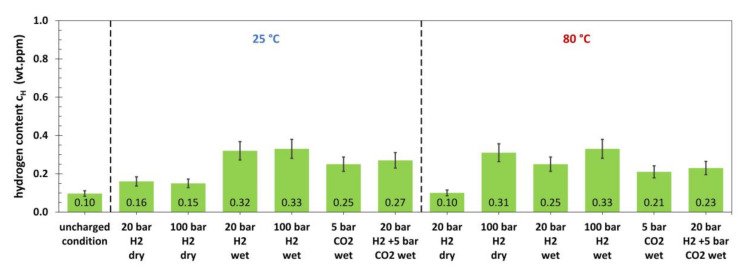
Hydrogen content of L80 after 30 days of autoclave testing with various media at 25 °C and 80 °C (wet conditions: with electrolyte; 15,000 ppm Cl^−^).

Results for the hydrogen content of immersion specimens made of L80, which were tested in autoclaves under increased pressure, are given in Figure 4.

**Figure 5:**
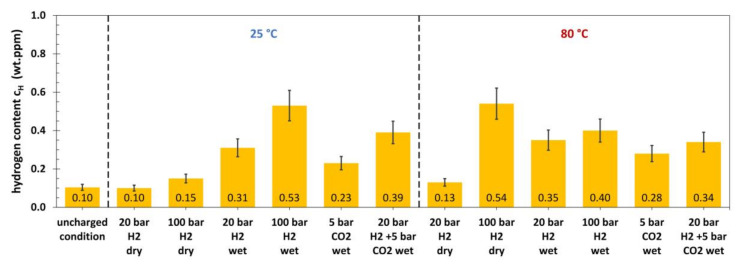
Hydrogen content of 42CrMo4 after 30 days of autoclave testing with various media at 25 °C and 80 °C (wet conditions: with electrolyte; 15,000 ppm Cl^−^).

In the uncharged condition, a hydrogen content of 0.10 wt. ppm was measured. Dry H_2_ gas with a pressure of 20 and 100 bar did not lead to significant amounts of absorbed hydrogen at 25 °C. At 80 °C, 20 bar dry H_2_ gas was not sufficient to cause a higher hydrogen content than in the uncharged state, whereas a total hydrogen content of 0.31 wt. ppm was measured after charging in 100 bar. The presence of brine (wet conditions) promoted the hydrogen absorption in high pressure H_2_ gas atmospheres at both temperatures tested and a maximum hydrogen content of 0.33 wt. ppm was measured. CO_2_ gas increased the hydrogen content of the immersion specimens without the presence of H_2_ gas and a maximum of 0.25 wt. ppm was found after testing at 25 °C. The mixture of the two gases resulted in a hydrogen uptake that was slightly above the level of the conditions with only CO_2_ gas. Figure 5 shows the measured hydrogen content for the 42CrMo4 after autoclave testing.

**Figure 6:**
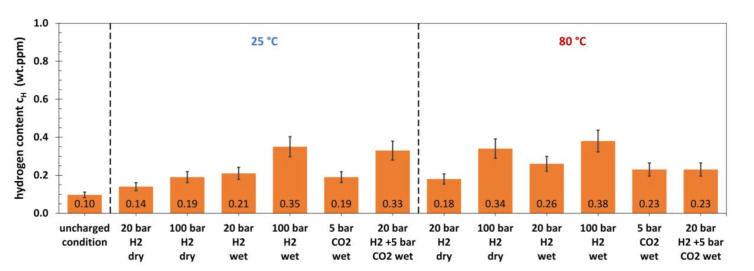
Hydrogen content of P110 after 30 days of autoclave testing with various media at 25 °C and 80 °C (wet conditions: with electrolyte; 15,000 ppm Cl^−^).

Like for the L80, the hydrogen content of 42CrMo4 (Figure 5) in the uncharged condition was 0.10 wt. ppm. At 25 °C, dry H_2_ gas with a pressure of 20 and 100 bar did not cause any significant hydrogen absorption. The same was true for 20 bar dry H_2_ gas at 80 °C, whereas 100 bar resulted in a total hydrogen content of 0.54 wt. ppm. Wet conditions with H_2_ gas promoted hydrogen absorption at 25 and 80 °C. The hydrogen content measured after autoclave tests on 42CrMo4 with CO_2_ gas was comparable to the results obtained for the L80 under the same conditions. The gas mixture also clearly caused an increase in hydrogen absorption. The results for P110 are shown in Figure 6.

**Figure 7:**
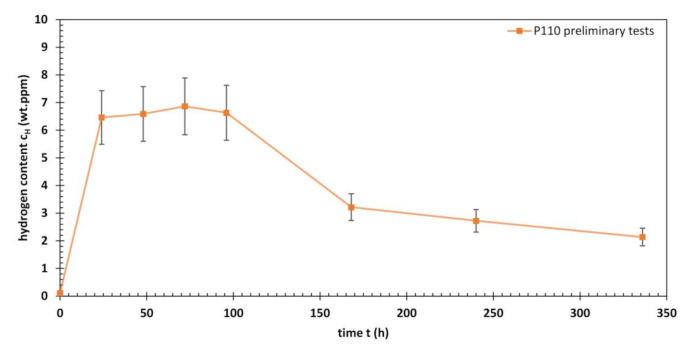
Hydrogen content of P110 after various times of ambient pressure testing in an acidified H_2_S-saturated aqueous brine solution with 5.0 wt.% NaCl and 0.5 wt.% CH_3_COOH at room temperature (NACE Solution A).

**Figure 8:**
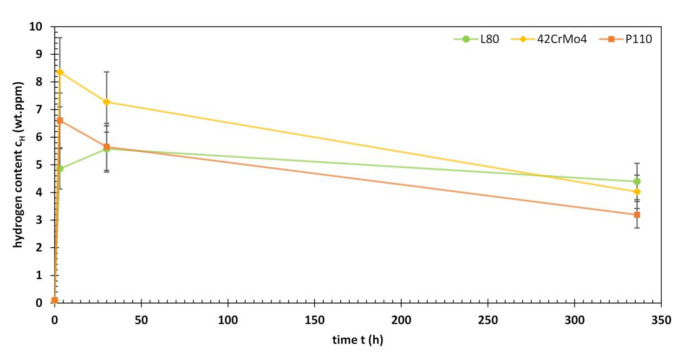
Hydrogen content of L80, 42CrMo4 and P110 after 3, 30 and 336 h of ambient pressure testing in an acidified H_2_S-saturated aqueous brine solution with 5.0 wt.% NaCl and 0.5 wt.% CH_3_COOH at room temperature (NACE Solution A).

Without charging, the P110 had a total hydrogen content of 0.10 wt. ppm, which corresponds to the other two materials tested. Of the dry conditions, only the combination of 100 bar H_2_ gas and 80 °C resulted in a hydrogen content that was significantly higher than that of the uncharged condition, while the others at least resulted in an increased content. This behaviour is similar to that of L80 and 42CrMo4. The combination of electrolyte and H_2_ gas promoted the hydrogen absorption at both temperatures tested and a maximum hydrogen content of 0.38 wt. ppm was measured. CO_2_ gas also increased the hydrogen content of the P110 immersion specimens, as was the case with the other two materials tested. The mixture of the two gases caused an increase in hydrogen absorption, this behaviour being much more pronounced at 25 °C than at 80 °C. Figure 7 and Figure 8 show the results of the hydrogen analyses on specimens charged at ambient pressure.

When steel is immersed in an acidified H_2_S-saturated aqueous brine solution, the conditions of hydrogen charging may change over time due to the formation of a corrosion product layer and slight variations in pH. H_2_S tests were conducted on similar specimens made of P110 to determine the hydrogen content as a function of time. The results of these tests are shown in Figure 7. The hydrogen content shows a plateau during the first days of immersion in NACE Solution A and a decrease towards the end of the test. This behaviour can be explained by the formation of a corrosion product layer. The scale consisting of iron sulfides reduces the further corrosion rate and thus the number of hydrogen atoms generated for possible absorption. The layer may not reduce hydrogen effusion to the same extent. The results for the hydrogen content of L80, 42CrMo4 and P110 after various times of ambient pressure testing in acidified H_2_S-saturated aqueous brine solution are shown in Figure 8.

Prior to the immersion in NACE Solution A, all three materials tested had a hydrogen content of 0.10 wt. ppm. After three hours of testing, the first specimens were removed from the solution and hydrogen analyses indicated significant amounts of absorbed hydrogen. The total hydrogen contents for L80, 42CrMo4 and P110 were 4.86, 8.35 and 6.61 wt. ppm. In the following 27 h, the value for L80 further increased to 5.58 wt. ppm, while those for 42CrMo4 and P110 decreased to 7.27 and 5.65 wt. ppm. At the end of the test (336 h) a hydrogen content of 4.40, 4.03 and 3.19 wt. ppm was measured for L80, 42CrMo4 and P110. A look at the shape of the curves shown in Figure 8 reveals a similarity between those for 42CrMo4 and P110, the latter is shifted to lower hydrogen contents. The maximum hydrogen content for both materials was measured after three hours of H_2_S testing. The L80 with its maximum after 30 h contrasts this trend.

### 3.2. Constant Load Tests

**Figure 9:**
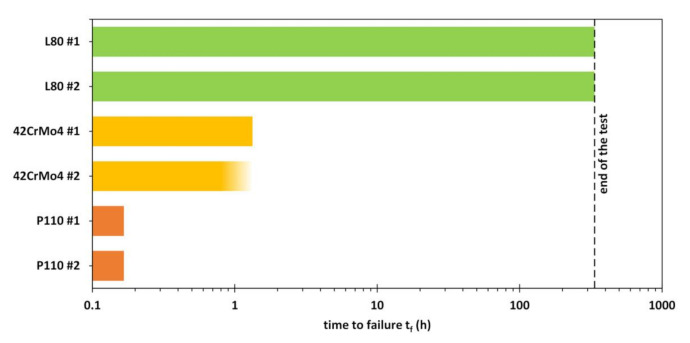
Results of the CLTs at 90% of the SMYS in acidified H_2_S-saturated aqueous brine with 5.0 wt.% NaCl and 0.5 wt.% CH_3_COOH at room temperature (NACE Solution A). The bar with a blurred end represents a failure where time is not exactly known.

None of the specimens constantly loaded at 90% of the SMYS broke under the conditions tested in the autoclaves (up to 100 bar H_2_ gas). None of the unbroken specimens showed visible cracks under the stereo microscope. Figure 9 shows the results of the CLTs in NACE Solution A at ambient pressure. The dashed line represents the end of the test after 14 days.

The first specimens to fail in H_2_S were those made of P110. These were broken after only ten minutes of immersion. They were followed by the 42CrMo4, failing after slightly less than 80 min and after 80 min respectively. None of the specimens made of L80 failed in NACE Solution A during the 336 h of testing.

## 4. Discussion

The hydrogen analyses on specimens tested in autoclaves gave results, which indicate an overall low but still detectable hydrogen absorption. The values for hydrogen uptake presented in this work are in good agreement with findings from the literature where similar steel grades were charged in H_2_ gas [21,37,38,42,43]. Fluctuations in the total hydrogen content after autoclave tests under different conditions can be attributed to different causes. According to Sieverts and Krumbhaar [4] the partial pressure of hydrogen gas has a strong influence on the amount of hydrogen absorbed by iron. This dependency was found for to all three steels tested, being most pronounced in dry conditions at 80 °C.

The presence of electrolyte promoted hydrogen uptake from H_2_ gas at 25 °C. Since the specimens still had a metallic lustre after the test, the influence of corrosion on the hydrogen absorption can be neglected. Nevertheless, the specimens tested under wet conditions with H_2_ gas looked slightly tarnished and therefore some corrosion may have occurred. With 20 bar H_2_ gas at 80 °C the presence of brine also led to a higher hydrogen content of all tested materials. Under the same conditions, with the exception of a higher pressure (100 bar H_2_), this behaviour was not found for the 42CrMo4, while L80 and P110 behaved the same way as already observed at 20 bar. An explanation for the comparatively high hydrogen content (0.54 wt. ppm) of 42CrMo4 after autoclave testing with 100 bar dry H_2_ gas at 80 °C cannot be given. It is assumed that this slightly higher content compared to 100 bar wet H_2_ lies within the scatter of hydrogen analysis.

Conditions with CO_2_ gas resulted in uniform corrosion of the immersion specimens. A dark grey scale was observed. It can be concluded from the cleaning of additionally tested specimens in hydrochloric acid that the layer consisted of iron(II) carbonate FeCO_3_ (siderite). The CO_2_ corrosion led to hydrogen absorption. The addition of 20 bar H_2_ gas further increased the hydrogen content. Although corrosion already is considered critical regarding hydrogen embrittlement, 100 bar H_2_ gas can lead to even higher hydrogen contents than corrosive environments with CO_2_ gas.

At 80 °C, the investigated materials tend to absorb more hydrogen, when exposed to dry H_2_ gas than at 25 °C. This observation is in line with Sieverts’ law, according to which the hydrogen solubility in metals increases with temperature. Under wet conditions, additional factors may influence the temperature dependence of the amount of hydrogen absorbed. The solubility of gases in brine generally decreases with temperature while chemical reaction rates (e.g., in the event of corrosion) double with each 10 °C. The temperature dependence of the hydrogen uptake under conditions with CO_2_ gas may be influenced by a change in morphology of the corrosion product film. Above 65 °C a more protective corrosion product layer is formed [62]. Overall, the results show no significant temperature influence on the hydrogen absorption of the carbon steels that were tested in autoclaves under wet conditions.

The hydrogen contents measured after the ambient pressure tests with H_2_S were significantly higher than those found in the autoclave tests without H_2_S. Corrosion and inhibition of recombination of hydrogen atoms to H_2_ molecules due to H_2_S promoted hydrogen absorption. Initially higher corrosion rates and formation of a somewhat protective iron sulfide layer during the immersion led to high hydrogen contents at the beginning of the test with a decrease towards the end. In general, the amount of hydrogen absorbed under conditions with H_2_S is approximately one order of magnitude larger than under conditions with up to 100 bar H_2_ gas.

A higher defect density and the increase in strength leads to a higher number of hydrogen traps. Regarding the maximum hydrogen content measured after autoclave tests with H_2_ gas as well as ambient pressure tests with H_2_S, the higher strength P110 absorbed more hydrogen than the lower strength L80. The 42CrMo4 has the same UTS and even a lower YS than the P110 but absorbed slightly more hydrogen during both types of tests. This behaviour can be explained by the somewhat finer microstructure of 42CrMo4, which offers more interfaces that serve as possible hydrogen traps. Since the volume fraction of retained austenite in the microstructure of all three materials tested is less than 1%, its influence on the trapping of hydrogen can be neglected.

In ambient pressure tests with H_2_S for the materials 42CrMo4 and P110 the maximum hydrogen content was measured after three hours. The L80 with its maximum measured after 30 h seems to be lagging behind. A possible explanation could be a slower corrosion rate with an associated lower hydrogen generation. Occasional visual inspections during the experiment have indeed shown that the formation of the corrosion product layer on the L80 was slightly retarded compared to the other materials. This effect could be attributed to fewer defects in the lower strength material.

The hydrogen content reached in the autoclave tests with up to 100 bar H_2_ gas was not sufficient to cause hydrogen embrittlement leading to failures of tensile specimens under a constant load of 90% of the SMYS. Ambient pressure conditions with H_2_S resulted in a failure of P110 after only ten minutes of immersion. Since a hydrogen content of 6.61 wt. ppm was measured 170 min after the failure in H_2_S and 0.38 wt. ppm was not sufficient for a failure in H_2_ gas, the threshold value for hydrogen embrittlement lies somewhere in between. This is in agreement with the findings of Asahi et al. [20], where a critical hydrogen content of 1.3 wt. ppm is proposed for the P110. Simultaneously, the threshold for 42CrMo4 is between 0.54 wt. ppm and 8.35 wt. ppm. The equation postulated by Asahi et al. [20] can be used to calculate a limit of 8.2 wt. ppm for this material.

**Figure 10:**
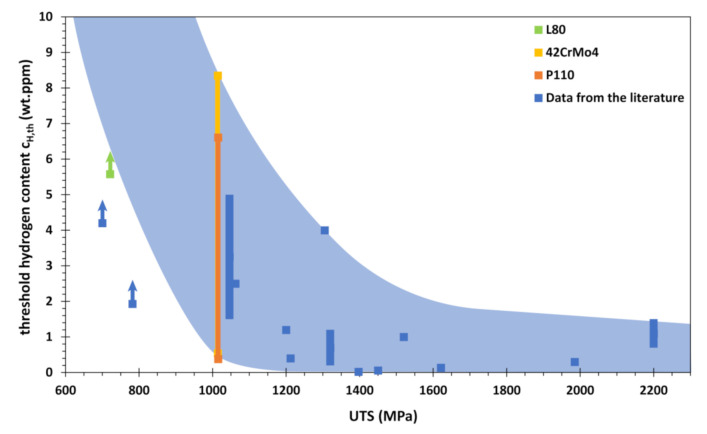
Threshold hydrogen content for hydrogen embrittlement of various martensitic and bainitic steel grades. Literature data from [19,20,21,22,23,24,42,43,44,52,53,54], missing values for UTS were estimated with YS × 1.10.

Since the specimens made from L80 did not fail under a constant load of 90% of the SMYS, although a maximum hydrogen content of 5.58 wt. ppm was found, the threshold concentration for hydrogen embrittlement is greater than this value. This is in accordance with the findings from the literature, where a critical hydrogen content of 22.8 wt. ppm was proposed [20]. In the present work, the maximum hydrogen content measured after immersion in up 100 bar H_2_ gas was 0.33 wt. ppm and thus significantly lower. The sour service steel grade L80 is therefore suitable for application under the tested conditions with up to 100 bar H_2_ gas. The critical hydrogen contents for embrittlement found in this work are compared with data from the literature in Figure 10.

## 5. Conclusions

The following conclusions can be drawn from the present work:

- H_2_ gas with a pressure of 20 and 100 bar results in an overall low hydrogen absorption. An increase of the partial pressure leads to a higher hydrogen content. Under dry gas conditions an increase in temperature results in a higher hydrogen absorption. The presence of electrolyte promotes hydrogen uptake from H_2_ gas at 25 °C.
- The hydrogen content achieved in autoclave tests with 100 bar H_2_ gas does not cause any substantial hydrogen embrittlement in specimens made from L80, 42CrMo4 and P110 under a constant load of 90% of the SMYS.
- The amount of hydrogen absorbed under conditions with H_2_S is approximately one order of magnitude larger than for conditions with up to 100 bar H_2_ gas. The high hydrogen content leads to failures due to hydrogen embrittlement for 42CrMo4 and P110 at 90% of the SMYS. The sour service grade L80 does not crack during 14 days of immersion in H_2_S-saturated solution.
- Material strength and microstructure play a crucial role in the extent of hydrogen absorption and embrittlement.
- The threshold hydrogen content for hydrogen embrittlement under a constant load of 90% of the SMYS lies between 0.38 wt. ppm and 6.61 wt. ppm for the P110, between 0.54 and 8.35 wt. ppm for the 42CrMo4 and above 5.58 wt. ppm for the L80. This is in accordance with findings from the literature.
- The sour service steel grade L80 is suitable for application under the tested conditions with up to 100 bar H_2_ gas.

## Figures and Tables

**Table 1:** Chemical composition of the investigated materials (wt.%).

Material	C	Si	Mn	P	S	Cu	Cr	Ni	Mo	Fe
L80	0.33	0.21	1.38	0.017	0.009	0.02	0.25	0.02	0.01	bal.
42CrMo4	0.42	0.27	0.85	0.014	0.012	0.01	1.01	0.02	0.17	bal.
P110	0.31	0.21	1.36	0.011	0.007	0.02	0.24	0.02	0.01	bal.

**Table 2:** Specified minimum yield strength (SMYS), yield strength (YS), ultimate tensile strength (UTS) and fracture elongation (A) of the investigated materials.

Material	SMYS (MPa)	SMYS (ksi)	YS (MPa)	YS (ksi)	UTS (MPa)	UTS (ksi)	A (%)
L80	552	80	607	88	721	105	17.3
42CrMo4	750	109	765	111	1014	147	12.1
P110	758	110	921	134	1015	147	8.4
